# Complex phylogeny and gene expression patterns of members of the NITRATE TRANSPORTER 1/PEPTIDE TRANSPORTER family (NPF) in wheat

**DOI:** 10.1093/jxb/eru231

**Published:** 2014-06-09

**Authors:** Peter Buchner, Malcolm J. Hawkesford

**Affiliations:** Rothamsted Research, Plant Biology and Crop Science Department, West Common, Harpenden, Hertfordshire AL5 2JQ, UK

**Keywords:** *Triticum aestivum*, nitrate, transport, nutrition, senescence.

## Abstract

NPF (formerly referred to as low-affinity NRT1) and ‘high-affinity’ NRT2 nitrate transporter genes are involved in nitrate uptake by the root, and transport and distribution of nitrate within the plant. The NPF gene family consists of 53 members in *Arabidopsis thaliana*, however only 11 of these have been functionally characterized. Although homologous genes have been identified in genomes of different plant species including some cereals, there is little information available for wheat (*Triticum aestivum*). Sixteen genes were identified in wheat homologous to characterized *Arabidopsis* low-affinity nitrate transporter NPF genes, suggesting a complex wheat NPF gene family. The regulation of wheat NFP genes by plant N-status indicated involvement of these transporters in substrate transport in relation to N-metabolism. The complex expression pattern in relation to tissue specificity, nitrate availability and senescence may be associated with the complex growth patterns of wheat depending on sink/source demands, as well as remobilization during grain filling.

## Introduction

Along with the initial uptake of nitrate by the root, the distribution of nitrate and N-containing metabolites between different organs and tissues is a complex process influenced by growth and development of a plant. For cereal crop production, one of the most critical stages is post-anthesis grain filling, involving both *de novo* uptake from the soil and remobilization of previously acquired N. In wheat and barley, up to 90% of the nitrogen is remobilized from the vegetative plant parts to the grain (Gregersen *et al.*, 2008). The importance of nitrate transporter genes in determining overall efficiency is not well characterized.

Nitrate transporters have been best characterized in the model plant *Arabidopsis*. Plant nitrate transporters are divided into the NPF (former ‘low-affinity’ NRT1/PTR) and ‘high-affinity’ NRT2 gene families (Tsay *et al.*, 2007; Léran *et al.*, 2014). The *Arabidopsis* genome contains 53 NPF genes and 7 NRT2 genes (Tsay *et al.*, 2007). The *Arabidopsis* NRT2 gene family is well characterized; most of the NRT2 genes are expressed in the root with the exception of *AtNRT2.4* and *AtNRT2.5*, which are also expressed in leaves, and *AtNRT2.7*, which seems to be leaf-specific (Orsel *et al.*, 2002). Mutant analysis of most NRT2 genes in *Arabidopsis* indicates involvement in nitrate transport (Cerezo *et al.*, 2001; Wang *et al.*, 2012). Gene expression of many NRT2 genes is regulated by nitrate availability and other factors (Zhuo *et al.*, 1999; Orsel *et al.* 2002; Orsel *et al.*, 2006).

Functional ‘high-affinity’ nitrate transport requires a second protein, Nar2, together forming a two-component nitrate uptake system (Zhou *et al.*, 2000; Tong *et al.*, 2006; Orsel *et al.*, 2006).

As the NRT1/PTR gene family encompasses transporters for different molecules, including nitrate, peptides, amino acids, auxins, abscisic acid and glucosinolates (Tsay *et al.*, 2007; Nour-Eldin *et al.*, 2012; Krouk *et al.*, 2010; Kanno *et al.*, 2012; Chen *et al.*, 2012), it is termed the NPF (‘NRT1-PTR-Family’). Phylogenetic analysis indicates eight subfamilies numbered NPF1–NPF8 (Léran *et al.*, 2014). With the exception of AtNPF6.3, which is a dual-affinity (low and high) nitrate transporter (Liu *et al.*, 1999), most of the NPF transporters characterized are ‘low affinity’. *AtNPF6.3* is expressed predominantly in the root (Tsay *et al.*, 2007) and is regulated by the N-status, but it is also expressed in guard cells (Guo *et al.*, 2003) and influences stomatal function. AtNPF6.3 has bidirectional transport activities involved in root-to-shoot nitrate translocation (Léran *et al.*, 2013). In addition to root dual-affinity nitrate uptake, AtNPF6.3 also functions as a nitrate sensor (Ho *et al.*, 2009) with the switch between functions regulated by phosphorylation (Liu and Tsay, 2003; Ho *et al.*, 2009). The nitrate signalling function of AtNPF6.3 is crucial for root architecture, influencing lateral root development (Forde, 2002). The control of lateral root development seems to be based on the dual nitrate/auxin transport activity, whereby high nitrate concentrations suppress auxin transport, which promotes lateral root development, and vice versa (Krouk *et al.*, 2010). AtNPF4.6 is also involved in low-affinity root nitrate uptake. In contrast to *AtNPF6.3*, which has reduced expression under N-starvation and is inducible by nitrate resupply (Tsay *et al.*, 1993), *AtNPF4.6* is constitutively expressed.

Other *Arabidopsis* NPF transporters seem to be involved in the distribution of nitrate within the plant. For example, after uptake, the first step is loading nitrate into xylem vessels for root-to-shoot nitrate translocation, and AtNPF7.3 may fulfil this role (Lin *et al.*, 2008). Furthermore *NFP7.3 Arabidopsis* mutants have reduced nitrate content in the xylem sap with reduced nitrate translocation to the shoots, supporting the role of NFP7.3 in root nitrate xylem loading (Lin *et al.*, 2008). Two other NFP transporters, AtNPF7.2 and AtNPF2.9, may be involved in root-to-shoot nitrate translocation; in contrast to *AtNPF7.3*, *AtNPF7.2* and *AtNPF2.9* mutants showed enhanced root-to-shoot nitrate transport. The plasma membrane-located AtNPF7.2 is expressed mainly in the xylem parenchyma cells of roots, indicating a function in removing nitrate from the xylem back into the root cells (Li *et al.*, 2010). Expression of *AtNPF2.9* was detected in companion cells of the root phloem (Wang and Tsay, 2011). In *Arabidopsis*, two genes, *At5G62680* and *At3G47960*, are phylogenetically closely related to AtNPF2.9. Functional analysis revealed that both transporters are capable of transporting glucosinolates; both gene products AtNPF2.10 and AtNPF2.11 have been shown to be plasma membrane located with cellular localization in adjacent mesophyll cells and leaf veins, respectively. Mutant analysis indicates a role in the transport of glucosinolates from source tissues to the grain (Nour-Eldin *et al.* 2012). The *Arabidopsis* NPF transporters AtNPF6.2 and AtNPF2.13 may have a role in leaf distribution of nitrate: *AtNPF6.2* is mainly expressed in the leaf petioles and mutation of *AtNPF6.2* reduces the nitrate content in the petiole and increases the nitrate content in the leaf lamina (Chiu *et al.*, 2004). *AtNPF2.13* is expressed in the phloem of older leaves and may be important for the source-to-sink remobilization of nitrate by allocation of nitrate from older to younger leaves (Fan *et al.*, 2009). Recent data suggested that NPF1.1 and NPF1.2 are involved in xylem-to-phloem transfer to enable redistribution of nitrate into developing leaves, a critical step for optimal plant growth (Hsu and Tsay, 2013).

The plasma membrane-localized *AtNPF2.12* is exclusively expressed in the vascular bundle of the siliques, suggesting involvement in nitrate transport to the developing seeds (Almagro *et al.*, 2008).

Nitrogen remobilization during senescence in cereals is an important factor influencing grain quality and yields, however the extent to which NPF transporters determine the efficiency of these processes is unknown. In the rice (*Oryza sativa*) genome there are at least 80 genes belonging to the NPF family but few have been characterized. Modern wheat varieties have a high degree of nutrient use efficiency, and nitrogen mobilization from old leaves to new leaves and into the grain contribute to efficiency. This report identifies and characterizes the wheat NPF gene orthologues to the well characterized selected *Arabidopsis* NRT1 genes. Gene expression studies in relation to tissue specificity, N-status of wheat plants, as well as post-anthesis leaf senescence provides a first insight into the complex composition of wheat members of the NPF transporter gene family.

## Materials and methods

### Identification of wheat NFP genes in the wheat genome and phylogenetic analysis

Gene and mRNA sequences of the orthologous and phylogenetically closely related *Brachypodium distachyon* and rice sequences (Table 1) were used in a Blast analysis (Altschul *et al.*, 1997; EMBL/NCBI; DFCI Gene index; Graingenes; CerealsDB; IWGSC survey sequence). The genomic exon-intron structures and chromosome localizations were identified by alignment of the derived sequences to the CerealsDB and the IWGSC survey sequence databases (the original CerealsDB sequence reads are archived in SRA of the European Nucleotide Archive (ENA) with Study ID ERP000319). For wheat sequence accessions where only incomplete coding regions have been identified, the complete coding sequence and the genomic structure were derived by genomic blast walking and assembling using the wheat genome CerealsDB (for NRT1 D-genome sequences see Supplementary Table S1) and IWGSC wheat genome survey sequence databases.

**Table 1.** Symbol numbers and genome gene ID of selected *Arabidopsis thaliana*, *Brachypodium distachyon* and *Oryza sativa* (ssp japonica cv Nipponbare apart from OsNPF6.7 for which the ssp Indica annotation number was used) NFP genes

*Arabidopsis thaliana*		*Brachypodium distachyon*		*Oryza sativa*	
Symbol	TAIR Gene ID	Symbol	Brachypodium.org Gene ID	Symbol	MSU Gene ID
AtNPF6.3	At1g12110	BdNPF6.3 BdNPF6.4 BdNPF6.5 BdNPF6.6	Bradi3g16670 Bradi1g78330 Bradi3g33040 Bradi3g33030	OsNPF6.3 OsNPF6.5 OsNPF6.4	Loc_Os08g05910 Loc_Os10g40600 Loc_Os03g01290
AtNPF4.6	At1g69850	BdNPF4.11	Bradi1g37330	OsNPF4.11	Loc_Os06g38294
AtNPF6.4	At3g21670	BdNPF6.7	Bradi3g47010	OsNPF6.7 OsNPF6.6	Os02g35830 Loc_Os04g39030
AtNPF6.2	At2g26690	BdNPF6.2	Bradi2g41060	OsNPF6.2	Loc_Os01g37590
AtNPF7.3	At1g32450	BdNPF7.10 BdNPF7.11	Bradi3g52096 Bradi3g53380	OsNPF7.9 OsNPF7.10 OsNPF7.11	Loc_Os02g46460 Loc_Os06g21900 Loc_Os02g48570
AtNPF2.12	At1g27080	N/A	N/A	N/A	N/A
AtNPF2.13	At1g69870	BdNPF2.6	Bradi2g58470	OsNPF2.5	Loc_Os01g68510
AtNPF7.2	At4g21680	N/A	N/A	N/A	N/A
AtNPF2.9A	At1g18880	BdNPF2.4	Bradi4g00530	OsNPF2.2 OsNPF2.3	Loc_Os12g44100 Loc_Os12g44110
AtNPF2.10	At3g47960	N/A	N/A	N/A	N/A
AtNPF2.11	At5g62680	N/A	N/A	N/A	N/A
AtNPF1.2	At1g52190	BdNPF1.2	Bradi2g50580	OsNPF1.2	Loc_Os01g55610

Phylogenetic analysis was performed by multiple protein sequence alignment using ClustalX V. 2.1 (Larkin *et al.*, 2007). MEGA 5.05 (Tamura *et al.*, 2011) was used for calculation of phylogenetic trees [the neighbour-joining method (Saitou and Nei, 1987)]. Bootstrap values for the trees were calculated as a percentage of 1000 trials with a seed number for the random number generator of 1000 (Felsenstein, 1985). The evolutionary distances (expressed as number of amino acid differences per site) used the number of differences method (Nei and Kumar, 2000).

### Plant material

The plant material for total RNA isolation was from hydroponically grown wheat (cv. Paragon), and from field-grown wheat (cv. Hereward) with 200kg N ha^–1^ (as ammonium nitrate) in triplicate repetition [Stackyard field (medium loam) at Rothamsted Research, Harpenden, UK, in 2006/7]. In field trials, the wheat varieties cv. Paragon and cv. Hereward exhibit similar characteristics in relation to N-uptake, N-remobilisation and grain N-utilization efficiencies (Barraclough *et al.*, 2010; Barraclough *et al.*, 2014). The spring wheat, cv. Paragon, requiring no vernalization, was used for all hydroponic experiments and the winter wheat cv. Hereward in field trials.

For the N-induction experiment, wheat was grown hydroponically in a modified Letcombe nutrient solution [1.5mM Ca(NO_3_)_2_, 5mM KNO_3_, 2mM NaNO_3_, 1mM MgSO_4_, 1mM KH_2_PO_4_, 25 μM FeEDTA, 160nM Cu(NO_3_)_2_, 9.2μM H_3_BO_3_, 3.6 µM MnCl_2_, 16nM Na_2_MoO_4_, 5 µM KCl and 770nM ZnCl_2_; Drew and Saker, 1984] under sufficient nitrate supply in a growth chamber and 16h/8h light/dark daily cycle. Nutrient solutions were exchanged three times a week. Two weeks after germination, plants (apart from the +N control plants) were N-starved for 1 week by replacement of nitrate salts with the corresponding chloride salt. After 1 week of N-starvation, N-induction was initiated by placing N-starved plants back into full nitrate nutrient solution (apart from –N control plants). Samples were taken at day 0, 3, 5 and 8 (+N) and at day 3, 5 and 8 (–N), and 30min, 1h, 2h, 4h, 8h and 24h after nitrate induction. Roots were washed, dried and frozen immediately in liquid nitrogen. Whole shoots were harvested, frozen immediately in liquid nitrogen, and stored at –80°C.

For field-grown wheat, the second leaf below the ear from 10 primary shoots per plot was harvested from anthesis, weekly until complete senescence. The leaves were frozen in liquid nitrogen and stored at –80°C.

The relative chlorophyll content was monitored in the middle part of at least 10 leaves using a Soil Plant Analysis Development (SPAD) meter (SPAD-502, Minolta, Japan) to monitor senescence. All plant materials were homogenized using a SPEX freezer mill (SPEX CertiPrep Ltd, UK) in liquid nitrogen, aliquoted into 2ml micro-tubes and stored at –80°C.

### Plant total RNA isolation

Total RNA was isolated by a modified method based on Verwoerd *et al.* (1989) including additional phenol-chloroform-isoamyl alcohol extractions. Possible genomic DNA contamination was removed by RNase-free DNase treatment. The final air-dried pellet was dissolved in an appropriate volume of RNase-free water.

### Nitrate content analysis

Ground, freeze-dried root and shoot samples (20–30mg sample^–1^) were extracted at 80°C in de-ionised water. After centrifugation and passage through a 0.2 µm filter, nitrate was measured on a Skalar Continuous Flow Analyser (Skalar SAN^PLUS^ System, Skalar, UK).

### Reverse transcriptase – real-time PCR transcript analysis

Gene expression was analysed by quantitative and relative real-time PCR. First-strand cDNA synthesis was performed from 2 µg total RNA and dT-adapter primer (Invitrogen Superscript III; standard protocol, 2h synthesis time). Real-time PCR was performed using the Applied Biosystems 7500 Real Time PCR System and the SYBR^®^ Green JumpStart^™^ Taq ReadyMix^™^ (Sigma-Aldrich, UK). The 25 μl reactions contained 1 µl cDNA and 250nM of each primer. Partly degenerated primer combinations were used to cover gene expression of the genes from all three wheat genomes (Supplementary Table S2). The primer Tm and PCR conditions should allow binding of the primer as long as the wheat variety sequences analysed differed by one or two base sequence polymorphisms. Due to the sequence similarity of TaNPF2.4/2.5, a useful primer for real-time PCR could only be generated for the amplification of both isoform transcripts. Primer efficiency was analysed and only primer combinations were used with primer efficiencies between 85 and 115%. For greater accuracy, the mean primer efficiency was estimated using the linear phase of all individual reaction amplification curves (Ramakers *et al.*, 2003) calculated by using the LinRegPCR package (Tuomi *et al.*, 2010). By comparison of different constitutive normalization control wheat genes the Actin 3 gene showed the best performance and was used for the normalized relative quantification of expression. The normalized relative quantity (NRQ) of expression was calculated in relation to the CT values and the primer efficiency (E) of the target gene (X) and the normalizing reference gene (N) as Normalized Relative Expression (NRE) based on Rieu and Powers, 2009:

$$\text{NRE }=\;{\left({\text{E}}_{\text{X}}\right)}^{–\text{CT},\text{ X}}/{\left({\text{E}}_{\text{N}}\right)}^{–\text{CT},\text{ N}}$$

Statistically significant changes in relation to N-starvation/N-induction and time course of harvests were calculated by analysis of variance (ANOVA) of the log_2_-transformed NRE data (Gomez and Gomez, 1984).

For verification of transcripts, approximately 500-bp long cDNA-derived PCR fragments (Supplementary Table S3), genomic PCR fragments (for nonexpressed NRT1 genes), and corresponding real-time PCR fragments (data not shown) of the individual NRT1 genes were cloned into pGEM-Teasy (Promega, UK), sequenced (Eurofins, Germany), and submitted to EMBL (Supplementary Table S3). For each NPF gene analysed, quantitative real-time SYBR green PCR expression analysis in young roots and shoots was performed including a standard dilution series of each plasmid-PCR fragment in triplicate. Based on the molecular weight of plasmid and PCR fragments, the mRNA copy number per μl cDNA was calculated after actin normalization of CT values. The significance of differences in transcript copy number was analysed by ANOVA.

## Results

### Phylogenetic relationships of the putative wheat low-affinity nitrate transporters

In *Arabidopsis*, 12 NPF transporter genes are well characterized (Table 1) with functional data for nitrate transport activity (for review, see Wang *et al.*, 2012; Tsay *et al.*, 2007; Léran *et al.*, 2014). By sequence database BLAST analysis, orthologous full-length sequences and other phylogenetically closely related wheat NPF genes and their coding regions were identified (Table 2; Table S1 for all CerealsDB and IWGSC survey D-genome sequences). The symbol numbering used follows that suggested by Léran *et al.*, 2014.

**Table 2.** Wheat NPF and other wheat gene (analysed by gene expression) accession numbers of databases sequences

Wheat gene symbol	IWGSC Wheat chromosome localization	Accession numbers
TaNPF1.1	3AL/B/DL	BQ806518;TaAffx.52263.1.S1_at;HF545002
TaNPF2.1	5AS/BS/DS	AK334628;TC280584;HF544999
TaNPF2.2	5AS/BS/DS	BJ244453;CV780655;BJ250261;HF545000
TaNPF2.3	2AS/BS/DS	HF545001
TaNPF2.4	3AL/B/DL	TC400476;HF544994
TaNPF2.5	3AL/B/DL	HF544995
TaNPF4.1	7AL/BL/DL	CA732431;CK207315;TC432669;HF544989
TaNPF6.1	7AL/BL/DL	AK333802;HF544985
TaNPF6.2	1AL/BL/DL	BJ279931;TC401315;GH722017;HF544986
TaNPF6.3	1AL/BL/DL	BG907608;TC391493;HF544987
TaNPF6.4	5AL/4BS/4DL	HF544988
TaNPF6.5	1AS/BS/DS	AK330268;HF544991
TaNPF6.6	5AL/BL/DL	AK332369;HF544990
TaNPF6.7	2AL/BL/DL	HF545004
TaNPF7.1	6AL/BL/DL	BJ279017;HX175323; TC380559;HF544992
TaNPF7.2	6AL/BL/DL	TC334619;BU099863;TC433104;HF544993
TaActin (3 like)	5AL/BL/DL	Ta.28253.1.S1_at;TC441720
TaSAG12	2AL/BL/DL	AB267407;TC232300
TaRubiscoSSU	5AL/BL/DL	Ta.27923.2.S1_x_a;TC263601
TaGS1	6AL/BL/DL	DQ124209;DQ124210;DQ124211
TaGS2	2AL/BL/DL	DQ124212;DQ124213;DQ124214
TaGSe	4AS/BS/DS	AY491970;AY491971
TaGSr	4AS/BS/DS	AY491968;AY491969
TaGDH2	2AL/BL/DL	AK331666;TC266053
TaNR1	6AS/DS/ BS?	TC236448;Ta.5633.1.S1_at;AL825459;AK333426
TaNAM	6AS/BS/DS	DQ869673;DQ869672;DQ869675
TaNIR	6DL/AL?/BL?	FJ527909;TC392193; HM989894

**Fig. 1.**
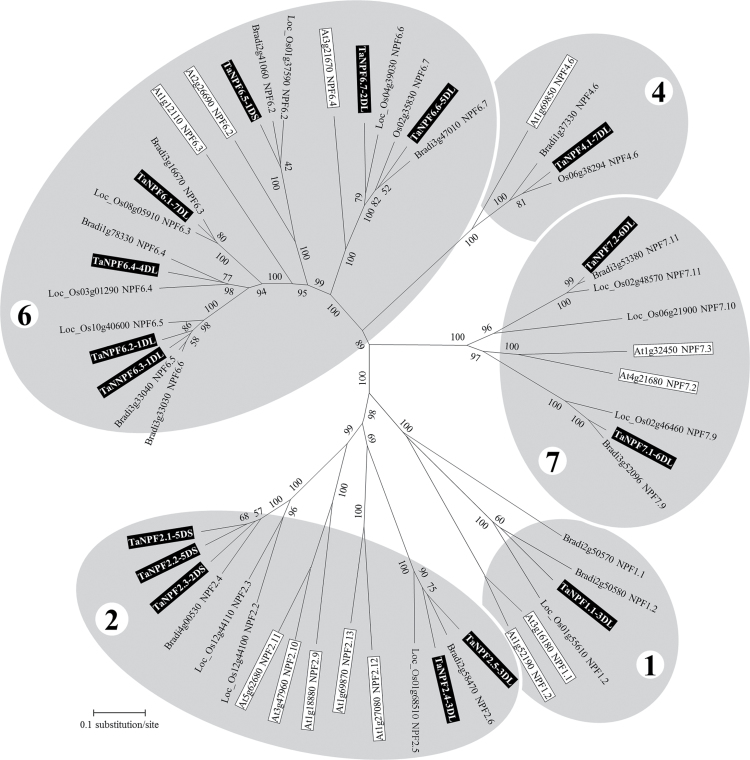
Evolutionary radial relationships of the wheat NPF proteins to related NPF proteins from *Arabidopsis thaliana*, *Oryza sativa*, and *Brachypodium distachyon* (gene ID Table 1). Neighbour-joining evolutionary tree analyses (Saitou and Nei, 1987) were conducted in Mega5 (Tamura *et al.*, 2011) from the multiple alignment (ClustalX V.2.1; Larkin *et al.*, 2007) of the proteins of selected *Triticum aestivum*, *A. thaliana*, *O. sativa*, (ssp. japonica cv. Nipponbare, a part of OsNPF6.7 for which the ssp. Indica annotation number was used), and *B. distachyon* NPF genes. The TAIR genome, MSU, and Brachyposium.org IDs were used for the *Arabidopsis* (square frame, white highlighting), rice, and *Brachypodium* sequences, respectively, including the NPF numbering. For simplification only D-genome-located wheat protein sequences (square frame, black highlighting) were used. The chromosome number and chromosome arm location (short-S; long-L) follows the NPF numbering. Each group number (grey areas) represents the NPF subfamily number. Bootstrap values were calculated as a percentage of 1000 trials with a seed number for the random number generator of 1000 (Felsenstein, 1985). The evolutionary distances (number of amino acid differences per site) used the number of differences method (Nei and Kumar, 2000). The analysis involved 56 protein sequences. All positions containing gaps and missing data were eliminated. There was a total of 314 positions in the final dataset.

Phylogenetic analysis of the 16 identified wheat NPF D-genome proteins in comparison to the *Arabidopsis*, *Brachypodium* and rice orthologous and homologous proteins (gene ID Table 1) showed that they can be classified into the NPF subfamilies 1, 2, 4, 6 and 7 (Fig. 1 – group numbers for grey areas; Léran *et al.*, 2014). As in *Brachypodium*, four wheat homologous proteins, TaNPF6.1, TaNPF6.2, TaNPF6.3, and TaNPF6.4, were identified as co-orthologous to *Arabidopsis* AtNPF6.3. The orthologous wheat *NPF6.2* and *NPF6.3* genes are both located on the long arm of chromosome 1D. Although TaNPF6.1, as with the *Brachypodium* and rice NPF6.3 proteins, has the shortest phylogenetic distance to *Arabidopsis* NPF6.3 compared to the other three co-orthologous proteins, a clear orthologous relationship was not verified, in agreement with Plett *et al.*, 2010. In the *Brachypodium* genome, as in *Arabidopsis*, only one *NPF6.4* orthologous gene exists. In wheat, two co-orthologous genes, *TaNPF6.6* and *TaNPF6.7*, were identified in the D-genome, and they are closely related to the orthologous genes *OsNPF6.6* and *OsNPF6.7* in rice. Both wheat genes are located on different chromosomes (Fig. 1). As in rice and *Brachypodium*, only one gene orthologous to *AtNPF6.2*, *TaNPF6.5*, was identified in the wheat D-genome (Fig. 1).

*AtNPF4.6* of subfamily 4 is represented in wheat as well as in rice and *Brachypodium* by one orthologous gene, *TaNPF4.1*.

The *Arabidopsis* proteins AtNPF7.3 and AtNPF7.2 are phylogenetically closely related and located in subfamily 7 (Fig. 1). In wheat, two genes were identified which are phylogenetically closely related. Both wheat genes are orthologous to *Brachypodium BradiNPF7.9* and *BradiNPF7.11* and the three rice genes *OsNPF7.9*, *OsNPF7.10*, and *OsNPF7.11* (Fig. 1). The phylogenetic distance of the TaNPF7.1 protein is closer to both *Arabidopsis* proteins than the TaNPF7.2 protein, but as already described in Plett *et al.* (2010), an orthologous relationship is not clear.

The *Arabidopsis* proteins for NPF2.12, NPF2.13, NPF2.9, NPF2.10, and NPF2.11 belong to the NPF subfamily 2 (Fig. 1). The wheat TaNPF2.4 and TaNPF2.5 proteins are phylogenetically related to AtNPF2.12 and AtNPF2.13 from *Arabidopsis*. Plett *et al.* (2010) suggested that *Brachypodium* BradiNPF2.6 and rice OsNPF2.5 are homologous to *Arabidopsis* AtNPF2.13, and that there may be no homologous/orthologous gene in either species related to *Arabidopsis AtNPF2.12*. Both TaNPF2.4 and TaNPF2.5 proteins from wheat are closely related to BradiNPF2.6, which suggests that there are two co-orthologous genes in wheat. The location of both genes on the long arm of chromosome 3D suggests gene duplication (Fig. 1).

Plett *et al.* (2010) did not include the *Arabidopsis* NPF genes for *At1g18880* (*AtNPF2.9*), *At3g47960* (*AtNPF2.*10) and *At5g62680* (*AtNPF2.11*). Tsay *et al.* (2007) reported nitrate transporter activity for the AtNPF2.9 and AtNPF2.11 gene products. Further analysis by Nour-Eldin *et al.* (2012) indicated glucosinolate transporting capability for both AtNPF2.10 and AtNPF2.11. In the *Brachypodium* genome, only one gene, *Bradi4g00530 (BradiNPF2.4)*, showed a close phylogenetic relationship with the *Arabidopsis NPF2.9*, *NPF2.10*, and *NPF2.11* group. On chromosome 12 of the rice genome, there are two neighbouring genes, *Os12g44100* and *Os12g44110*, whose gene products have the closest phylogenetic relationships. In the wheat D-genome there are three genes phylogenetically closely related to *Arabidopsis NPF2.9*, *NPF2.10* and *NPF2.11*. Both *TaNPF2.1* and *TaNPF2.2* are located on the short arm of chromosome 5D, suggesting gene duplication, and *TaeNPF2.3* is located on the short arm of chromosome 2D (Fig. 1).

The wheat NPF1.1 protein is similar to *Brachypodium* BradiNPF1.2 and rice OsNPF1.2, and all are orthologous to *Arabidopsis* NPF1.1 and NPF1.2 (Fig.1).

### Quantitative gene expression of NPF in roots and shoots of wheat seedlings

Profiles of the expression patterns in the root and shoot of young wheat plants 3 weeks after germination and grown hydroponically under sufficient nitrate nutrition (+N control plants of the N-starvation experiment) provide the first information on tissue specificity or dominance of the individual NPF transporters.

**Fig. 2.**
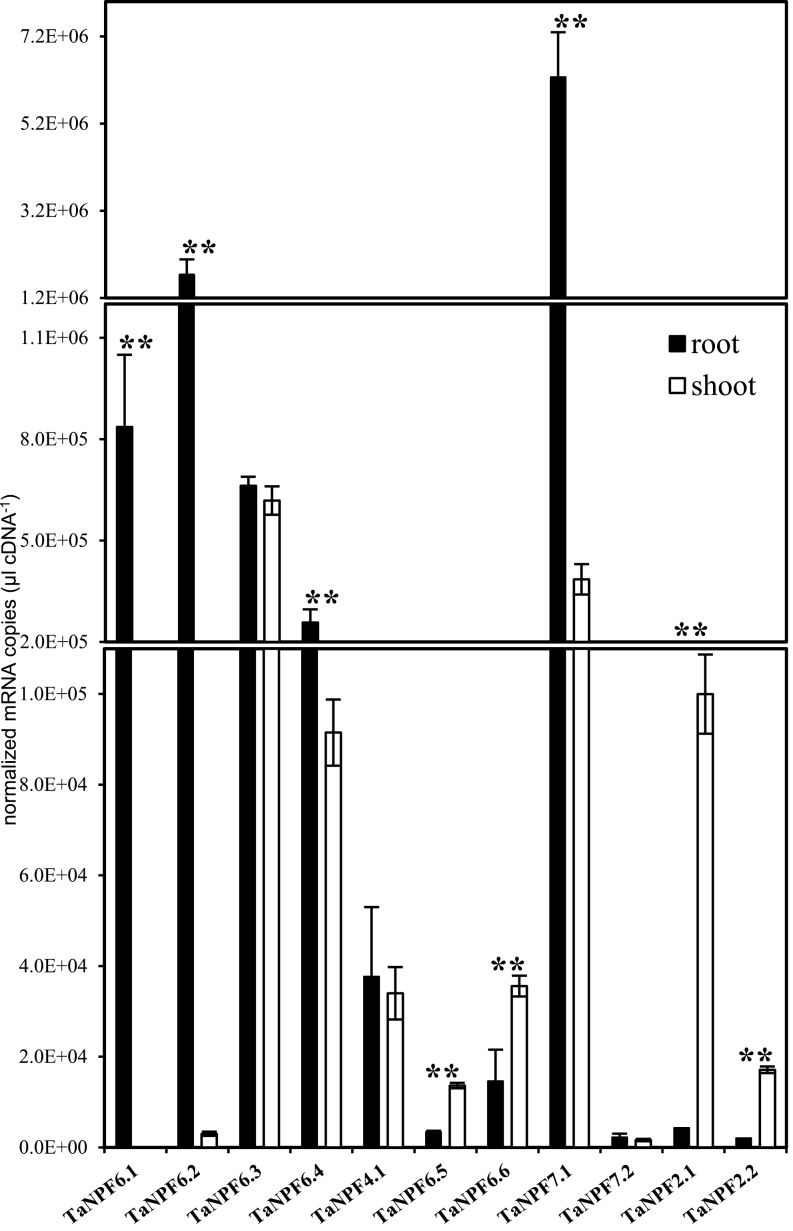
Quantitative real-time PCR expression analysis of putative low-affinity nitrate transporters TaNPF6.1, TaNPF6.2, TaNPF6.3, TaNPF6.4, TaNPF4.1, TaNPF6.5, TaNPF6.6, TaNPF7.1, TaNPF7.2, TaNPF2.1, and TaNPF2.2 in roots and shoots of young hydroponically grown wheat plants. Each bar represents the mean ±SE of at least three biological replicates. **, significant differences between root and shoot (*P* < 0.01) of individual NPF genes.

Expression of the four wheat genes NPF6.1 to 6.4, co-orthologous to AtNPF6.3, in the root and shoot of young wheat was distinctive. *TaNPF6.1* and *TaNPF6.2* transcripts were present with high abundance in the roots and very low abundance in the shoots (Fig. 2). The transcript abundance of *TaNPF6.3* was very similar in the roots and in the shoots, and *TaNPF6.4* transcript was significantly higher in the roots but also with reasonable transcript levels in shoots (Fig. 2).

As for *TaNPF6.3* and *TaNPF6.4,* the transcript level of *TaNPF4.1* was very similar in the roots and shoots (Fig. 2), but the overall transcript copy number was much lower.

*TaNPF7.1* showed the highest mRNA copy number in roots amongst all NPF genes analysed (Fig. 2). Although there was a dominant high transcript level of *TaNPF7.1* in roots, the shoot transcript level was still reasonably high, and similar to the shoot transcript abundances of *TaNPF6.3*, *TaNPF6.4* and *TaNPF2.1*. The transcript level of *TaNPF7.2* was very similar in roots and shoots, but was very low compared to *TaNPF7.1* (Fig. 2).

*TaNPF6.6*, *TaNPF6.5*, *TaNPF2.1* and *TaNPF2.2* were highly expressed in the shoot (Fig. 2). Whilst the root transcript level of *TaNPF6.6* was still around 50% of the shoot level, the root mRNA copy levels of *TaNPF6.5*, *TaNPF2.1* and *TaNPF2.2* were 25%, 12% and 4.3%, respectively, much lower compared to the shoot. Although in the wheat genome a third co-orthologous/homologous gene *TaNPF2.3* is present, so far no clear transcript could be detected.

### Influence of N-starvation and N-induction on the expression of NPF genes in roots and shoots

**Fig. 3.**
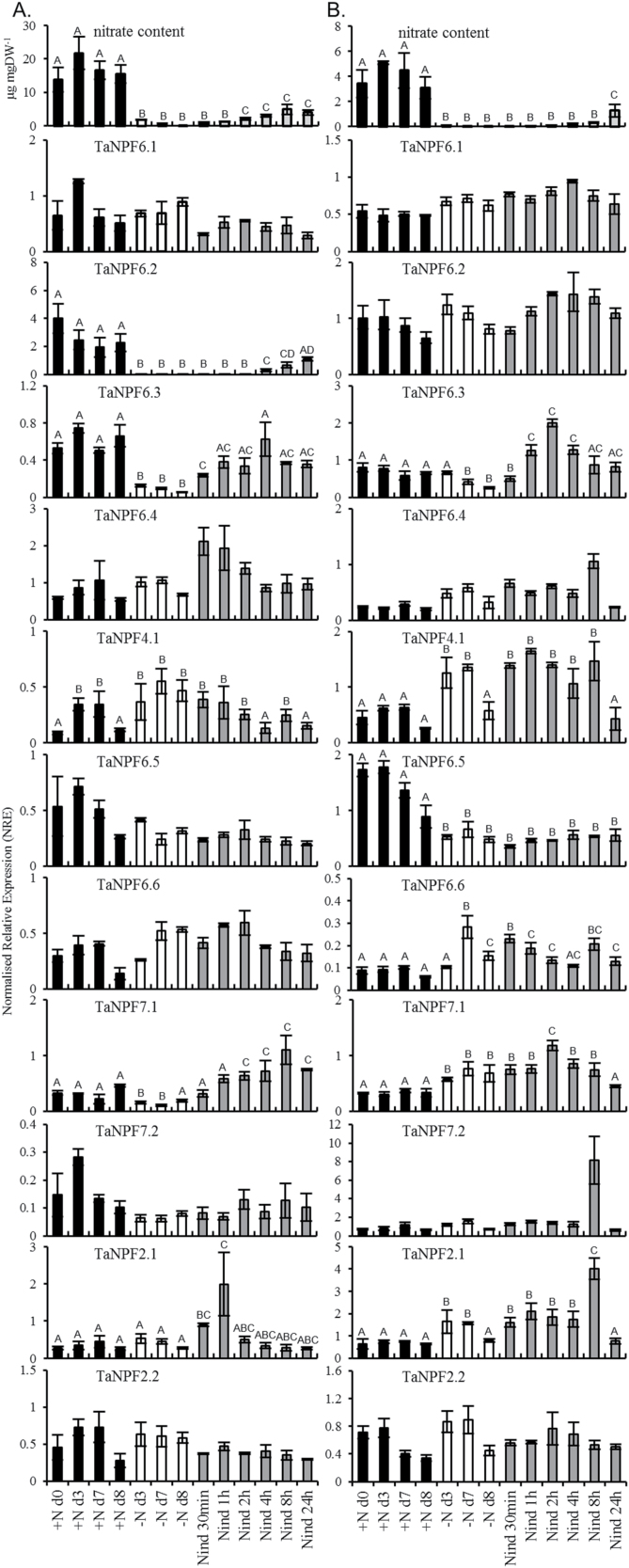
Influence of nitrate starvation and nitrate induction on the nitrate content and gene expression of wheat NPF genes in roots (A) and shoots (B) of young wheat plants. NRE, ‘Normalized Relative Expression’; black bars, + N treatment; white bars, – N-starvation; grey bars, nitrate induction. Each bar represents the mean ±SE of at least three biological replicates. Different letters on the top of the bars indicate significance of *P* < 0.05. Letters shared in common indicate no significant difference.

For some *Arabidopsis* NPF genes an influence of N-availability on gene expression has been reported (overview Wang *et al.*, 2012). Three days’ nitrate starvation-deprivation of hydroponically grown wheat plants reduced the nitrate content in roots to 10% and in shoots to less than 1% compared to the controls, with further reduction to nearly zero in both with continuing starvation (Fig. 3). Nitrate resupply after 7 days of starvation resulted in a significant increase of nitrate in roots after 2h with a further increase until 24h, but without recovering to control content. In shoots a significant nitrate increase was only found after 24h of nitrate resupply (Fig. 3). The four wheat genes co-orthologous to AtNPF6.3 showed different patterns of expression in relation to the N-supply. In roots, *TaNPF6.2* and *TaNPF6.3* gene expression was clearly influenced by the N-status of the plants. The transcript of *TaNPF6.2* was significantly reduced by N-starvation. After 3 days of N-starvation, only very low expression was detectable, and after nitrate resupply expression was induced very slowly, with a significant increase of expression 4h after nitrate induction and with a further increase until 24h after N-induction, reaching similar levels to control + nitrate culture (Fig. 3A). The expression of *TaNPF6.3* was less down-regulated in roots by N-starvation compared to *TaNPF6.2*. With N-induction, the transcript level of TaNPF6.2 was restored after 1h to the average level found under sufficient N-supply (Fig. 3A). No influence, either by N-starvation or by N-induction, was detectable on expression of *TaNPF6.1* and *TaNPF6.4* genes in roots (Fig. 3A).

The expression of *TaNPF7.1* was significantly reduced after 3 and 6 days of N-starvation. After nitrate resupply, the expression increased to the +N level within 30min with a further increase between 2h and 8h, significantly higher compared to the control culture, with a slight reduction after 24h (Fig. 3A).

The root expression of most of the other wheat NRT1 genes analysed was not influenced by nitrate starvation and nitrate induction (Fig 3A). *TaNPF4.1* and *TaNPF2.1* expression indicated non-N-supply related high variation with significant changes in the +N culture and/or N-starvation and N-induction (Fig. 3A).

There is little information about the influence of nitrate starvation and induction on the expression of NRT1 genes in vegetative shoot tissues. In *Arabidopsis* shoots, induction/up-regulation of gene expression was reported for *AtNPF6.4* and *AtNPF6.2* (Okamoto *et al.*, 2003). In young wheat shoots the expression of several NPF genes is influenced by the N-supply. As in roots, *TaNPF6.3* expression decreased in shoots with nitrate starvation, but much later compared to roots; however after 7 days of starvation the expression is reduced by around 50% compared to the initial/average level of the +N control culture (Fig. 3B). With nitrate resupply, *TaNPF6.3* expression increased gradually to a maximum peak at 2h, which subsequently reduced gradually within 6h to the control +N level.

The expression of *TaNPF4.1* was significantly up-regulated by nitrate starvation. With nitrate induction this higher expression level continued for almost 8h and decreased at 24h to the +N control level (Fig. 3B). With some minor differences a similar expression pattern was also found for *TaNPF6.6*, *TaNPF7.1* and *TaNPF2.1*. In contrast to *Arabidopsis*, nitrate starvation in wheat resulted in a reduction of *TaNPF6.5* expression. After 3 days of N-starvation, *TaNPF6.5* expression decreased significantly to around 30% of the +N control level. This lower expression did not recover during nitrate resupply, but the +N control expression also decreased at day 8, suggesting a possible additional developmental influence.

### Influence of post-anthesis senescence on the leaf expression of wheat NPF genes

**Fig. 4.**
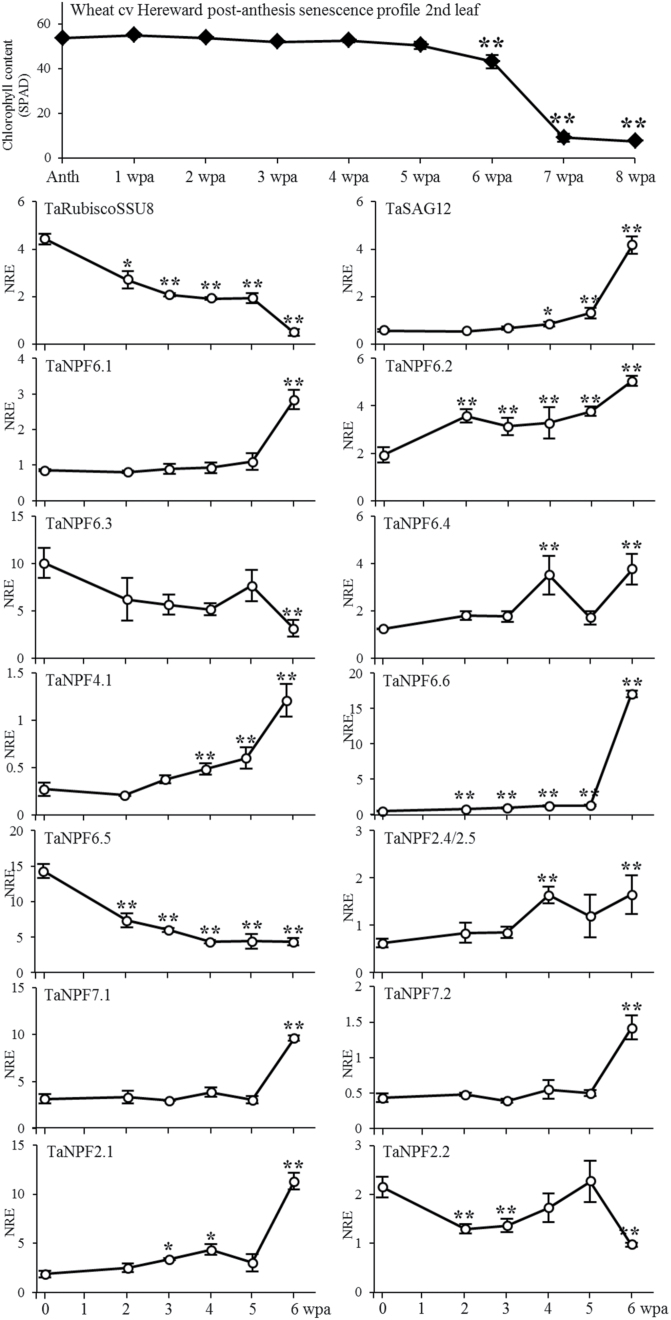
Expression analysis in relation to post-anthesis chlorophyll content/senescence in the second leaf of wheat. Real-time PCR expression analysis of ribulose bisphosphate carboxylase/oxygenase small subunit (RubiscoSSU on Chr5), senescence associated gene (SAG12), and putative low-affinity nitrate transporters NPF6.1, NPF6.2, NPF6.3, NPF6.4, NPF4.1, NPF6.5, NPF6.6, NPF7.1, NPF7.2, NPF2.4/2.5, NPF2.1, and NPF2.2. NRE, ‘Normalised Relative Expression’. Values are means ±SEM (n = 3). **, *, significantly different changes in time compared to anthesis (*P* < 0.01, *P* < 0.05, respectively). Anth, anthesis; wpa, weeks post-anthesis.

**Fig. 5.**
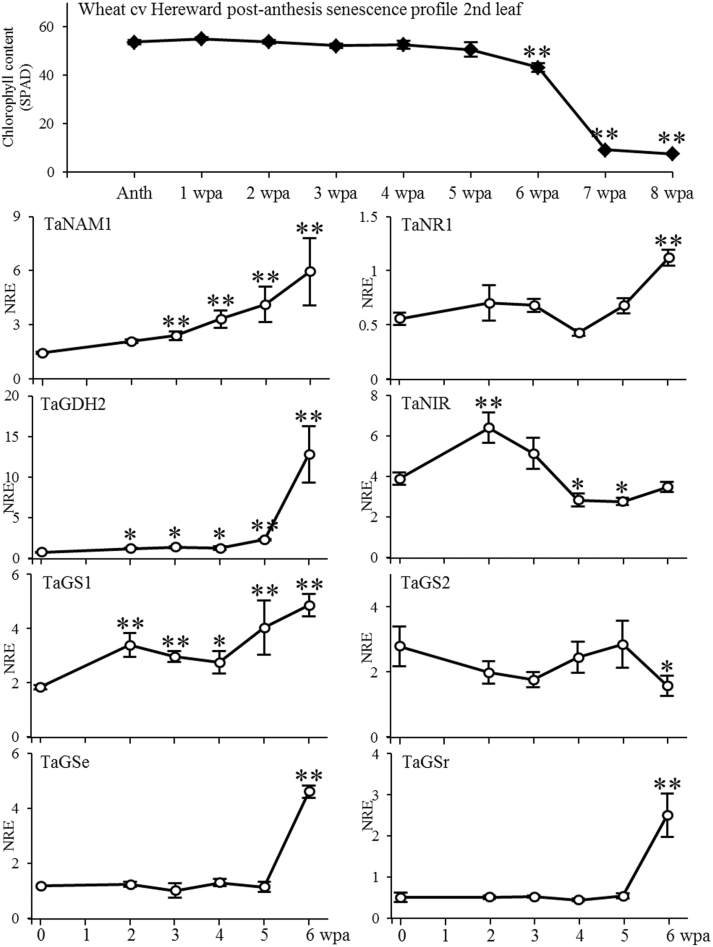
Expression analysis in relation to post-anthesis chlorophyll content/senescence in the second leaf of wheat. Real-time PCR expression analysis of NAC transcription factor (TaNAM), nitrate reductase 1 (TaNR1), nitrite reductase (TaNIR), glutamate dehydrogenase 2 (TaGDH2), glutamine synthetases TaGS1 (cytosolic), TaGS2 (plastidic), TaGse (cytosolic), and TaGSr (cytosolic). NRE, ‘Normalised Relative Expression’. Values are means ±SEM (n = 3). **, *, significantly different changes in time compared to anthesis (*P* < 0.01, *P* < 0.05, respectively). Anth, anthesis; wpa, weeks post-anthesis.

During wheat grain development the nitrogen in the vegetative tissues becomes an important N-reserve, as *de novo* root N uptake is insufficient for grain N demand and efficient re-translocation of N facilitated by shoot senescence is required (Foulkes *et al.*, 2009). Here data is provided for post-anthesis senescence and selected gene expression profiles for a single year (2006/07) field experiment (Figs 4 and 5). This year was representative of typical UK growing conditions when comparing climatic conditions for this year (see supplementary figure S1) to other years. The relative chlorophyll/senescence profile of the SPAD analysis of the middle part of leaf 2 indicates at least 50% senescence at 6 weeks post-anthesis, with reduction to less than 10% after a further 7 days (Figs 4 and 5). As a marker of senescence, gene expression levels of the small subunit of ribulose-1,5-bisphosphate carboxylase/oxygenase (RubiscoSSU) (Fig. 4), the expression profile of a wheat C1A cysteine protease (here called *TaSAG12*), orthologous to the *Arabidopsis* senescence-associated *AtSAG12* involved in protein degradation (Noh and Amasino, 1999) (Fig. 4) and the wheat NAC transcription factor TaNAM-B1, known to be involved in regulating senescence and nutrient remobilization from canopy to developing grains (Fig.5; Uauy *et al.*, 2006; Waters *et al.* 2009), were analysed. For *RubiscoSSU*, a reduction of relative expression compared to anthesis was already visible 2 weeks post-anthesis, remaining level for 3 weeks before decreasing to a low expression level 6 weeks post-anthesis (Fig. 4). *TaSAG12* expression increased 4 weeks after anthesis, at which time the relative chlorophyll content in the middle of the leaf was still not reduced, but leaf tips showed signs of senescence (data not shown). With senescence, *TaSAG12* expression increased eight-fold further (Fig. 4). An earlier increase of expression was found for TaNAM-B1 (Fig. 5). A significant increase of expression was detectable 3 weeks after anthesis with a continuous increase up to four times higher as compared to anthesis. The discrepancy between RubiscoSSU and TaSAG12/ TaNAM-B1 expression indicates that the process of leaf senescence and N-remobilization are independently regulated. Analysis of stay-green mutants has shown that in mutants with a defect in the chlorophyll catabolism pathway, soluble protein degradation during senescence may be close to normal, but light-harvesting and reaction centre thylakoid membrane proteins are much more stable (Thomas *et al.*, 2002).

For the majority of the NPF genes analysed, there was a strong correlation of expression in relation to leaf senescence. The post-anthesis relative expression levels of *TaNPF6.1*, *TaNPF6.6*, *TaNPF7.1*, *TaNPF7.2*, and *TaNPF2.1* were very similar with no change or only slightly increased transcript levels until 5 weeks post-anthesis, followed by a very strong increase at week 6, similarly to the *TaSAG12* expression (Fig 4). The increased expression was in the range of 2-fold higher (*TaNPF7.2*) up to 35-fold higher (*TaNPF6.6*). The transcript levels of *TaNPF6.2*, *TaNPF6.4*, *TaNPF4.1*, and *TaNPF2.4/2.5* increased much earlier, already at 3–4 weeks post-anthesis with a steady state higher level compared to anthesis, as found for *TaNPF6.2*, *TaNPF6.6* and *TaNPF2.4/2.5*, or further increasing, as found for *TaNPF4.1* (Fig. 4). For two NPF genes a post-anthesis reduction of the relative expression in leaf 2 was detected. The expression of *TaNPF6.3* remained level until week 5, before it decreased by 50% at week 6 in comparison to anthesis (Fig. 4). The *TaNPF2.2* transcript pattern was complex with a slight but significant reduction at 2 weeks post-anthesis, an increased expression at weeks 4 and 5, and a substantial decrease at 6 weeks post-anthesis (Fig. 4).

To put NPF expression in leaf 2 after anthesis in the context of nitrogen assimilation, gene expression patterns of nitrate reductase 1 (*TaNR1*), nitrite reductase (*TaNIR*), glutamate dehydrogenase 2 (*TaGDH2*), 3 cytosolic and 1 plastidic glutamine synthetases (*TaGS1*, *TaG2e*, *TaGSr*, and *TaGS2*, respectively) were analysed. A significant increase in relation to senescence was found for nitrate reductase TaNR1 transcript abundance at 6 weeks post-anthesis (Fig. 5). *TaNIR* gene expression increased slightly within 2 weeks and decreased back to the initial level with no further reduction until late senescence. Both cytosolic glutamine synthetase genes *TaGse* and *TaGSr* showed an expression pattern similar to some of the NPF genes with unchanged expression levels until week 5, and a drastic increase to a 3- to 5-fold higher level at 6 weeks post-anthesis (Fig. 5). The wheat glutamine synthetase *TaGS1* expression was similar to *TaNPF6.2* with increased post-anthesis expression at week 2, which stayed constant before a further increase at week 6 (Fig. 5). The glutamate dehydrogenase *TaGDH2* gene expression showed a nearly identical pattern compared to the *TaNPF6.6* gene, with a slow but significant steady increase in expression until week 5, which then drastically increased up to a 17-fold higher level (Fig. 5). The expression of *TaGS2* (plastidic protein localization) followed an opposite pattern by decreasing at week 6, as would be expected with increasing senescence (Fig. 5).

## Discussion

Expression studies of the wheat NPF genes identified in relation to tissue specificity, regulation by nitrate availability, and N-remobilisation in leaves during post-anthesis senescence indicate the functions of those genes.

### Complex phylogeny of wheat NPF genes in the wheat genome

Analyses of other plant transporter gene families have identified similarities between phylogenetic relationships and function (Buchner *et al.*, 2004; Takahashi *et al.*, 2012). All of the *Arabidopsis* NPF transporters for which wheat homologues have been identified in this study are able to transport nitrate, as verified by oocyte assays (Tsay *et al.*, 2007; Lin *et al.*, 2008; Fan *et al.*, 2009; Li *et al.*, 2010). Given the background knowledge that the orthologous *Arabidopsis* genes are somehow involved in nitrate uptake as well as transport/distribution within the plant, the phylogenetically close relationships would favour similar functions/ substrate transport specificities for the orthologous wheat genes.

Plett *et al.* (2010) identified orthologous and paralogous cereal NPF genes in the genomes of rice, *Sorghum*, maize (*Zea mays*) and *Brachypodium*. Phylogenetic analysis of the identified wheat key orthologous and homologous NPF proteins, in comparison to *Arabidopsis*, rice and *Brachypodium*, identified clear single orthologues only for *TaNPF4.1* and *NPF6.5*. For all other wheat NPF genes, orthologous genes to the related monocot species were identified, but as Plett *et al.* (2010) described, a clear analysis of orthologous relationships between the selected *Arabidopsis* NPF genes and the monocot species is not always possible. Plett *et al.* (2010) have shown that the NPF family structure in cereals seems to be much more complex than in *Arabidopsis* and other dicots. The differences found between wheat compared to rice and *Brachypodium* indicate a further complexity of the wheat NPF gene family and may be only partly explained by additional gene duplication and/or loss of genes during wheat evolution. The different gene composition for the NPF families in the different grass species may be explained by different evolutionary development influencing the genomes of the different grass species. The grass genomes differ in size, ploidy level and chromosome number. The hexaploid wheat genome (AABBDD; 2n=42) of ~16 000 Mbp size originated from two polyploidization events (Feldman *et al.*, 1995). The diploid rice genome (2n=24) is, with ~430 Mbp, much smaller than the wheat genome; the *Brachypodium* genome (2n=10) with ~272 Mbp is nearly 60 times smaller than the wheat genome. In general, the gene order in the nuclear genomes of all grasses has been preserved; nevertheless genomic rearrangements, duplication and polyploidization events occurred during the evolution of the different grass species leading to differences in gene copy number. For example, in comparison to its progenitors, hexaploid wheat seems to have deleted many low-copy DNA sequences since the polyploidization event (Feldman *et al.*, 1997). For rice, recent segmental duplications on Chromosomes 11 and 12 and massive ongoing individual gene duplications have been identified (Yu *et al.*, 2005). For wheat, at least 10 duplicated regions, which represent 67.5% of the genome, were identified, and Salse *et al.* (2008) suggested an ancient duplication of the diploid wheat genomes before their hybridization into polyploid wheat.

### Complex expression pattern of wheat NPF genes in different tissues and in relation to N-status of the plant

For most of the *Arabidopsis* NPF genes considered in this study, the expression pattern is closely related to their suggested function in relation to nitrate transport. With few exceptions, there is little available information on gene expression patterns of related cereal genes. The existence of co-orthologous genes in cereals and other plant species such as *Lotus japonicus* (Criscuolo *et al.*, 2012), in comparison to the *Arabidopsis* single NPF gene loci, suggest split and/or individual additional functions of those co-orthologous/homologous NPF genes. Based on the existence of co-orthologous genes, the regulation of those genes would enable a much higher variability in relation to nitrate uptake as well as distribution within the plant. A good example can be seen for *AtNPF6.3* in *Arabidopsis*. The four co-orthologous wheat NPF genes showed similar but also different patterns of expression in relation to tissue specificity and N-nutrition. The root dominance of *TaNPF6.1* and *TaNPF6.2* indicated a more specific function for nitrate uptake and/or translocation in the root. However, the expression patterns of the *NPF6.2* and *NPF6.3* genes in response to N-starvation and resupply suggest similar functions in relation to nitrate sensing, whereby the differences in the pattern in relation to the N-resupply response suggests an individual fine tuning of regulation. The regulation of *TaNPF6.3* by the N-supply in the shoot has so far only been reported for the orthologous NPF gene of *Brassica napus.* In contrast to *Arabidopsis*, shoot gene expression of the orthologous *Brassica* gene was negatively correlated with shoot nitrate concentration, whereas it was positively correlated with root nitrate concentrations (Le Ny *et al.*, 2013). This suggests an important specific function of TaNPF6.3 in regulation of nitrate distribution in wheat non-root tissues. This function is supplemented by a constitutive factor provided by the co-orthologous TaNPF6.4 protein.

The *Lotus japonicus* genome contains four genes co-orthologous to *AtNPF6.3*. Root expression analysis of two of these showed no induction by N-resupply after N-starvation, and the expression of one gene was even down-regulated (Criscuolo *et al.*, 2012). Lauter *et al.* (1996) reported that the *AtNPF6.3* co-orthologous tomato Soly*NPF6.*9 and Soly*NPF6.10* genes had root-specific expression. Whilst Soly*NPF6.10* root expression was nitrate inducible, the Soly*NPF6.9* expression behaved constitutively (Lauter *et al.*, 1996). Root expression analysis of two maize co-orthologous genes grown under low and adequate nitrate nutrition showed no response of either gene in relation to N-nutrition throughout the whole lifecycle (Garnett *et al.*, 2013). This emphasizes the complexity and variation in gene regulation in relation to nutrient uptake in crop plants with complex genomes, in comparison to a simple genome model plant such as *Arabidopsis*.

Those differences are not only related to multi co-orthologous genes. *TaNPF4.1*, the single orthologous gene to *AtNPF4.6*, is expressed in roots and shoots to a similar level, suggesting involvement in substrate transport in roots and in shoots. The orthologous *Arabidopsis NPF4.6* is described as a constitutive component of the low-affinity root nitrate uptake system (Okamoto *et al.*, 2003). Huang *et al.* (1999) reported root-specific expression and an involvement in root nitrate uptake, although promoter-GUS studies of NPF4.6 indicated expression in the vascular tissue of the root and hypocotyl and also in the stem and leaves (Kanno *et al.*, 2012). In contrast to *AtNPF4.6*, wheat *TaNPF4.1* shoot expression is influenced by the N-status of the plant. Difference in the regulation of *AtNPF4.6* orthologous genes was also reported for the *Lotus japonicus* orthologous gene. In contrast to *AtNPF4.6* and *TaNPF4.1*, the *Lotus japonicus* orthologous NPF gene expression in roots is up-regulated by nitrate resupply after N-starvation (Criscuolo *et al.*, 2012). These differences of orthologous gene expression in relation to tissue specificity and N-supply in different plant species suggest variation in the regulatory signalling pathways for those genes. This further suggests different functions for these orthologous NPFs in the different plant species in relation of substrate uptake and translocation in different tissues.

The demand for nitrate uptake by the root is driven by nitrate assimilation in the plant during development. Long-distance transport via the xylem, including uploading in the different parts of the plant, and also nitrate allocation between organs via the phloem, facilitates the distribution of nitrate and requires regulation. Several *Arabidopsis* NPF transporters are reported to be expressed in vascular tissues.

The expression pattern of the orthologous and phylogenetic close wheat NPF genes implicates similarities but also differences in relation to tissue specificity and N-nutrition compared to the related *Arabidopsis* NPF genes. The wheat *TaNPF7.1* expression had root dominance with the highest root transcript level of all NPF genes analysed indicating importance for root nitrate transport. Further, the shoot transcript level of *TaNPF7.1* was also still comparatively high (second highest) in relation to the low shoot expression described for *Arabidopsis AtNPF7.3*. In contrast to *Arabidopsis NPF7.3*, for which no regulation of shoot expression by the N-status was observed, the opposite regulation of *TaNPF7.1* expression by N-starvation and nitrate resupply in the root and the shoot suggests a participation of *TaNPF7.1*, not only in root nitrate xylem loading, but also in nitrate translocation in the shoot regulated by the N-status. As there is a low transcript level in both roots and shoots for *TaNPF7.2,* a similar function to *AtNPF7.2* in nitrate unloading from the xylem in the different tissues is possible.

Under stressed situations it has been observed that nitrate is reallocated to the root (Hernandez *et al.*, 1997). Li *et al.* (2010) found that the nitrate reallocation process is regulated by the induction of *NPF7.2* in *Arabidopsis*, which contributes essentially to Cd^2+^ stress tolerance. Furthermore it has been shown that, in contrast, *AtNPF7.3* is down-regulated in roots by various stresses. Under these conditions, nitrate is retained in roots, which contributes to stress tolerance in a similar mechanism to that proposed for the NPF7.2 nitrate reallocation (Li *et al.*, 2010; Chen *et al.*, 2012). As there is no orthologous NPF7.2 in grasses (Plett *et al.*, 2010), the up-regulation of the wheat *TaNPF7.1* in shoots under N-starvation stress may suggest a similar function to AtNPF7.2 by reallocation of nitrate in the shoot to the vascular system and finally back to the root and reduction of expression in the root under N-starvation to retain high nitrate concentration.

Three wheat genes are co-orthologous to *AtNPF2.9* but also closely related to the glucosinolate transporter *AtNPF2.10* and *AtNPF2.11*. None of the wheat homologous *NPF* genes showed dominance for root expression. In contrast they are expressed more dominantly in the shoots with very low transcript levels in the roots. Similar expression pattern with shoot versus root dominance was also reported for the orthologous rice *NPF2.2* gene (Ouyang *et al.*, 2010). In *Arabidopsis*, the root-dominant AtNPF2.9 may be responsible for loading of nitrate into root phloem, with the apoplastic nitrate source for NPF2.9-mediated loading coming from efflux of vascular parenchyma cells or leakage of the xylem stream (Wang and Tsay, 2011). If the orthologous cereal genes of rice and wheat are also expressed in the phloem, the dominant shoot expression would favour a function more in loading and/or unloading of nitrate to or from the shoot/leaf phloem. The regulation of *TaNPF2.1* expression by the N-status in the roots as well as in the shoots indicates that its function is controlled by the nitrate/nitrogen demand of the individual tissue.

Differences in expression patterns were also found for the wheat *NPF6.5* and *NPF6.6* genes. The orthologous *Arabidopsis AtNPF6.2* is strictly leaf specific with expression in the petioles and with a unique function in nitrate homoeostasis regulation (Chiu *et al.*, 2004). The wheat *TaNPF6.5* showed a dominant shoot expression but with significant detectable expression in the root, indicating an additional root function. The down-regulation of expression by N-starvation in the shoot suggests a similar participation of *TaNPF6.5* in regulation of nitrate homeostasis in non-root tissues. *TaNPF6.6* showed higher transcript levels in the shoot and reasonable expression in the root. Unlike the *Arabidopsis* orthologous *AtNPF6.4* gene, no up-regulation in the shoot or down-regulation in the root by nitrate resupply was observed for the orthologous *TaNPF* (Okamoto *et al.*, 2003). Similarly no influence of N-provision on the root expression was reported for the two *Lotus japonicus* orthologous genes (Criscuolo *et al.*, 2012). N-starvation resulted in a slow up-regulation of the wheat gene expression which went down slowly after nitrate resupply to the +N control level. Furthermore, *Arabidopsis AtNPF6.4* gene expression was also suppressed in the root by nitrate application (Okamoto *et al.*, 2003), which was not found for the wheat orthologues indicating a completely different regulation of *TaNPF6.6* compared to the *Arabidopsis AtNPF6.4* gene.

### Correlation of NPF gene expression to leaf senescence

For wheat and other annual cereal crops, senescence is linked to seed reproduction (Noodén 1988a, b). This control of senescence by reproduction is not found in *Arabidopsis*. The life-span of single *Arabidopsis* leaves is independent of the development of reproductive structures (Noodén and Penney, 2001). This implicates a completely different demand for regulation of nitrate uptake and distribution/remobilization in wheat compared to *Arabidopsis* during senescence and grain development. Post-anthesis N-remobilisation and translocation of pre-anthesis stored N-assimilates to support grain growth is an important part of wheat development. Between 51 and 91% of the grain nitrogen comes from vegetative N remobilisation of organic N in the form of free amino acids and macromolecules (Van Sanford and MacKown, 1987; Palta and Fillery, 1995). In addition to post-anthesis N-remobilisation *de novo* N-uptake accounts for 5–40% of the total grain N in wheat, with high variation between wheat varieties (Kichey *et al.*, 2007; Bogard *et al.*, 2010). Several components of the assimilatory pathway have been implicated in the remobilization processes. Protein as well as gene expression studies indicated increased levels of wheat glutamine synthetase GS1 and GSr, as well as glutamate dehydrogenase, and decreased level of GS2, in wheat leaves with increasing senescence (Kichey et. al., 2005; Bernard *et al.*, 2008). This could be confirmed by the analysis provided here (Fig. 5). Unusually there was an up-regulation of expression with senescence for the third cytosolic glutamine synthetase *TaGSe* gene, similarly to *TaGSr*. Additionally there was an up-regulation of the wheat nitrate reductase (*TaNR*) expression with senescence. During senescence-related N-remobilisation, the ammonia for glutamine synthesis can be provided by re-assimilation of ammonia released during protein hydrolysis, and by assimilation of nitrate via nitrate and nitrite reduction. In tobacco and tomato, nitrate reductase expression in leaves has been shown to be regulated by the nitrate content (Galangau *et al.*, 1988). In wheat a good correlation was found for post-anthesis N-uptake and nitrate reductase activity (Kichey *et al.*, 2007). The post-anthesis expression of NPF genes of different subfamilies in leaf 2 implicates a complex pattern of nitrate transport activity. The nitrate delivered by post-anthesis uptake needs to be taken up via the leaf vascular bundle and distributed within the leaf for further reduction and assimilation. The increased expression level of the wheat *NPF* genes with increasing senescence could be an indication of increased N-related substrate transport and accumulation. An increased level of nitrate would increase the demand for nitrate reduction requiring an increased expression of nitrate reductase. The expression level of the nitrite reductase at late senescence was the same as found at anthesis, and indicated continuing conversion of nitrite to ammonia. The senescence-correlated up-regulation of NPF genes in leaf 2 suggested two possible options: to enable direct transport of stored nitrate from the canopy tissue to the grain; or channelling of nitrate during senescence to nitrate/nitrite reduction to provide ammonium for glutamine synthetase. It is well established that N-assimilation to allow protein synthesis and leaf N remobilization occurs simultaneously. Therefore in addition to protein degradation during senescence, nitrate transport may be an important supportive part for the translocation process of N from senescing source tissues to provide sufficient N for grain filling influencing nitrogen use efficiency and yield. The NAM Nac-transcription factors are predicted to regulate genes that encode proteins that carry out physiological processes for nutrient remobilization and/or translocation to grain (Waters *et al.*, 2009). The fact that some nitrate transporters, such as TaNPF4.1, showed a similar expression pattern compared to the wheat NAM Nac-transcription factor, indicated a highly regulated process.

### Conclusion

The data presented for the selected wheat NPFs indicates the complexity of the phylogeny and expression of the 16 wheat NPF genes identified. The existence of multiple co-orthologous genes in wheat and other cereals as well as dicotyledonous crop plants, in comparison to *Arabidopsis*, suggests a higher complexity of pattern and regulation of N-related substrate uptake, translocation and redistribution compared to that described for *Arabidopsis*. The regulation of some of the analysed wheat NFP genes by N-supply suggested an involvement of those NFPs in substrate transport closely related to N-metabolism. An example is the existence of four orthologous NPF6.3 genes showing similarities and differences, in comparison the sole *Arabidopsis* NPF gene. The complexity of the expression pattern in relation to N-supply as well as N-remobilisation/leaf senescence suggests a requirement for a complex network of regulation of NPF gene expression in the process of N-uptake and distribution within the plant. This complexity described here for wheat and other important crop plants can be explained by the need to be able to adjust the nutrient uptake and distribution to a much more complex development including reproduction compared to the simple weed *Arabidopsis*. This demonstrates the limitation of simple model plants in generalising and adopting findings. Due to the broad range of substrates reported to be transported by NPF transporters, more detailed functional transport analysis and spatial tissue-specific expression patterns are required for a full understanding of the role of the NPF transporters in wheat.

## Supplementary material

Supplementary data can be found at *JXB* online.

Supplementary Table S1. CerealsDB sequence IDs.

Supplementary Table S2. Oligonucleotide primer sequences used for SYBR Green real time RT-PCR expression analysis.

Supplementary Table S3. Oligonucleotide primer sequences used for cDNA-PCR fragment cloning and sequencing including database accession.

Supplementary Figure S1. Average rainfall and max.-min. air temperature at Rothamsted field trails between 1 September 2006 and 31 August 2007.

Supplementary Data
