# Supplementary material for: Complex phylogeny and gene expression patterns of members of the NITRATE TRANSPORTER 1/PEPTIDE TRANSPORTER family (NPF) in wheat
Source: J Exp Bot. 2014 Jun 9;65(19):5697–710. doi: 10.1093/jxb/eru231 (PMC4176842; doi:10.1093/jxb/eru231)
Supplement: Supplementary Data [file supp_65_19_5697__index.html]

Complex phylogeny and gene expression patterns of members of the NITRATE TRANSPORTER 1/PEPTIDE TRANSPORTER family (NPF) in wheat — Supplementary Data 

# Complex phylogeny and gene expression patterns of members of the NITRATE TRANSPORTER 1/PEPTIDE TRANSPORTER family (NPF) in wheat

## Supplementary Data

Data files

**Files in this Data Supplement:**

- Supplementary Data - Supplementary Data
