# Supplementary material for: Complex phylogeny and gene expression patterns of members of the NITRATE TRANSPORTER 1/PEPTIDE TRANSPORTER family (NPF) in wheat
Source: J Exp Bot. 2014 Jun 9;65(19):5697–710. doi: 10.1093/jxb/eru231 (PMC4176842; doi:10.1093/jxb/eru231)

Complex phylogeny and gene expression patterns of members of the NITRATE TRANSPORTER 1/PEPTIDE TRANSPORTER family (NPF) in wheat

Peter Buchner and Malcolm J Hawkesford

Rothamsted Research, Plant Biology and Crop Science Department, West Common, Harpenden, Hertfordshire, AL5 2JQ, United Kingdom.

Supplemental material

Table S1. CerealsDB sequence IDs.

Wheat NPF	CerealsDB sequence IDs - unassembled 454 reads and Draft assembly of gene-rich regions used for assembling of coding and 5' and 3' non-coding sequences corresponding to D-genome	Chromosome localization
TaNPF1.1	F03ZQ2H02IRBTQ contig152180	3AL/ 3BL / 3DL
TaNPF2.1	GGSKSHR02GAAYS contig2418439 GKL6WMX02GHZMM GKJGB6C03CWDF4 GD37MD301AOQK0 GGQO11T01DL3YR GH8MDFQ01DC3AN GMB8IBH02JPPOX F6CI9PH02IP23B	5AS / 5BS / 5DS
TaNPF2.2	GKY3Q2G01EXDB2 F6PXRVY01B27VT F4JP4XR02H5O9 GH8MDFQ02CO4Y0 GG1VXPK05G6W41 F3QGDP101AP3LK GMC3H7L01DOL34 GGWF2QU01CDTA2	5AS / 5BS / 5DS
TaNPF2.3	contig1352322 GH95CX402F4230 GH8MDFQ05J4PHR GG7HVD103BFYN GKS5NH307GV6EH GG7JLI307GLPKV GKS7ENJ05JE9BW GDS1A9H01D4PAF	2AS / 2BS / 2DS
TaNPF2.4	GIB2VHM01AEDTP GHILVEO01COPML GKY3Q6201ECRO3 contig60637	3AL / 3BL / 3DL
TaNPF2.5	GJOJZ1301CR9VR contig30207 GEFGSVT01DIU96 GDHUJ9D02G60ZC F2S221K01B2H6G GKL6WN402IV0TB contig2722186 F01XQWW01BP4HD GGWFHOC01D8CGU	3AL / 3BL / 3DL
TaNPF4.1	GGSKSHR02HXVN1 contig205754 GG7HVD105FSFMQ F2HXLMV02H2BVY GKJDY9A01BCPZ6 GJ6U61T01AXWEJ GKL6WN401DZT9C	7AL / 7BL / 7DL
TaNPF6.1	F2SQ5YK02JG6IBn ; GKJH3DB06I3LR9 ; GD37MD302JXVII ; GHY6XPR08GFRUJ ; GKL6WN401EUJ2R ; GCSAYRC01ANI94 ; contig1039456 ; GDEB4RJ02JNVS5	7AL / 7BL / 7DL
TaNPF6.2	contig596501 GKK8K0002HS9C7 GINEZBA03BGKK3 contig2287968 F4H0DWW02H894P GKS7ENJ02DQ8PM GKS7ENJ02DQ8PM contig591497	1AL / 1BL / 1DL
TaNPF6.3	Contig161152 GKY3Q6202J0YM9 F6JU48R01B3ZPH GINDECP06IA5KX GGWH0HJ01CHX81 F2HXLMV02FX2Q1 contig1167925	1AL / 1BL / 1DL
TaNPF6.4	contig157382 GG1XCTX01BCEPJ GG1XLLD07G5TW6 GINDECP05GKGE7 contig414417 GKYNZJQ01AU0RD GIABLPU02ERUNP	5AL / 4BS / 4DL
TaNPF6.5	contig1269752 GJVZZDM02G4KOG GAOC5IM01ATWSW F62S5NF01DDFRB GIB2VHM01EL5HJ GCT78DC01CJE48 GINEZBA06GYR97 F4S2R7Z02IBMQN contig1225807	1AS / 1BS / 1DS
TaNPF6.6	contig1081440 GKY3Q6202JAVXM GHY6XPR02CYYTF GL5C2CV02GXJPD GKF76DR02GDW2A GMC3LKS02IO07I GDH717U01ATGLX GMEN2LX01CPOIV	2AL / 2BL / 2DL
TaNPF6.7	GIABLPU06IQONN GIOY55M01ADTR3 F1XBOI001AO6L4 contig1887750 contig1480399 GINDECP05ISYBI GJZO2FP02JSR65	5AL / 5BL /5DL
TaNPF7.1	GIPCY5M02H2312 GIOY55M01AGWBO GKJH3DB02CN9O6 GKS7ENJ06F6RMA F3THAEN01DMY0Y F6JU48R01B7NJZ GD9UPD201A6WXU GINEZBA06GOH0L GGWE0EB01EM0IR GH99TNM07IUN0P	6AL / 6BL / 6DL
TaNPF7.1	contig62756 GG1VXPK02CBVN9 F676MBK02IAGI3 F676MBK02IAGI3 GD4IG1U02JQ9GQ GJVZZDM01EBARQ GGQTHUK02FNAZM contig679982	6AL / 6BL / 6DL

Table S2. Oligonucleotide primer sequences used for SYBR Green real time RT-PCR expression analysis.

Gene symbol	***Forward primer***	***Reverse primer***
TaNPF2.1	CGG TGG TAC AGG TTC AAG AAG TC	CGG AGT ATA KGA AGC CCC AAA AG
TaNPF2.2	GTG GAC CTR TTC TAC CTT GTC AC	AGC CTT CTT GGG GCT TTC ATC G
TaNPF2.4/2.5	ACA ATG GAC TGT CAC CTT GGA ACA C	TGC AGT TAG GGC GAT TAA GGA TAT GG
TaNPF4.1	GCG CTC AAC TAC GTC TTC TTC TTG	CRA AAC CTC CAT AAA TTT ACC TTG CC
TaNPF6.1	CAA TCG GAC GGC CTT GAT TTC TTC	ACG TAG GCA CAC GTG GAC GAC
TaNPF6.2	ATC GAT GCA TGC TAC TTG CGS TTC	GCC ACC GAA TAC ACA CAA AAA CAA G
TaNPF6.3	CAY GGG AGC AAC GAC GGC TG	ATG CGT TTC TCC TTG TAC ACG TAG
TaNPF6.4	GAA CCT CGG TGA CGA TGG TGA TGA	AGC GGT GCC TTA ACC TCR ACA AG
TaNPF6.6	AGC AGG CRT CAR CCT CAA CAA TG	ATT GTC CAG AAG AGG ATG CAG GTG
TaNPF6.7	GCC GAC GAG AAA TGC TAG CTG G	CAC ATA CGG ACG TAC ATG GAA GC
TaNPF7.1	CTA CAA GAC CTG CGC CAT CTT C	GAT GAG GTA TAG CCG CGA GGA G
TaNPF7.2	CCC AGC AGT CAA AGC AAA CAC TG	GGA GGA ACA CCA CCA GGT TCA C
TaActin3 like	GAC GCA CAA CAG GTA TCG TGT TG	AGC GAG GTC AAG ACG AAG GAT G
TaRubiscoSSU8	GGT GGA GGA GGT CAA GAA GGA G	MGT CGT GAG TGA GCT GTT TAG GC
TaGS1	ATG ATC GCC GAG ACC ACC ATC C	TCG TCC AAA TCC TCC ART GGC C
TaGS2	GAA CAT GGA CCC GTA CAC CGT G	TSA GGT CCT TCA TAC CTT CAG CG
TaGSe	TCA CCT CCM TGA TCG CCG AGA	CAC ACA GAW RAC AGA ACC CAT CAA AG
TaGSr	GAC ATC AGG AGC AAA GCA AGG AC	GGA GCC GTC GTA GTT CCA CTT G
TaNR1	GGC CAA TTC YTT CAT CTC CTT CTG	TAC RTS CAC AGA TTG ATG CGT CSA
TaNIR	ACG AGG AGT AGG CCG GCT ASG AG	ATC AGC CGC AGC CCA TCT CTR C
TaNAM	CTA CAA GAA GAT CAA CAA GGC CGC	TCC ACG GAG TCC TCG CAC TC
TaGDH2	AGG ATG GGA GCA TTC ACC TTG G	GGA TAT AAG AAC TKT CAT CCA CCA CG

Table S3. Oligonucleotide primer sequences used for cDNA-PCR fragment cloning and sequencing including data base accession.

**Wheat Nitrate transporter symbol**	**Forward primer**	**Reverse primer**	**EMBL data base accession**
TaNPF1.1	GAT CCT GCC GGT GTA CAA GAA G	GGA GCC AGC TTT GTT GTG AAA GC	HF545002
TaNPF2.1	ATC ACC CTG CTC CAR CGG ATC	ATC ACC CTG CTC CAR CGG ATC	HF544999
TaNPF2.2	CAT GTA CGA CAG GTT CGT GGT G	TCG ASG TCR TCG CTG GCG TG	HF545000
TaNPF2.3	GAC GGC YAT GAT GTC ATG CTT YTG	AGG GYG TAT GTT GAT TCC CRA GG	HF545001
TaNPF2.4	GGT AGC AGG GCT TGT TGA ACA C	CTC TGC AGG ACA AAT TCC YTC SC	HF544994
TaNPF2.5	GGT AGC AGG GCT TGT TGA ACA C	TGT GCG GGA YAA ATA TCC CGT AC	HF544995
TaNPF4.1	GTS GAG GYG AAG CGC AAG AAC	GGC AAG GTA AAT TTA TGG AGG TTT YG	HF544989
TaNPF6.1	GTC TTC TTC GTC GGC TCC ATC	GAA CTT GTA GAG CTC GCC CTT G	HF544985
TaNPF6.2	TCT TCC TGT CCA TCG TCG GCA TG	AGA AGC GCA AGT AGC ATG CAT CG	HF544986
TaNPF6.3	TCG GCC TCT TCC TGT CCA TYG	GAG CTA CAC GTG TAT ACA CGG AC	HF544987
TaNPF6.4	GAT CAG GCT GCA ATA GTA GAG GTG	GAA CTG TGG CAC GAT GAG GAA G	HF544988
TaNPF6.5	CTG CGA GAA GAA GCG TCT CAC	CAC ATA CGG ACG TAC ATG GAA GC	HF544991
TaNPF6.6	GGA GCA GGC ATC ARC CTC AAC	GTG CTC ATG GAC TTC ATC CTC KC	HF544990
TaNPF6.7	TCC GCA CTC GTC GAG AAG AAA	CAA ACC CCA ACG CTG CCA ART AG	HF545004
TaNPF7.1	CGC SAC MAC CAT CTT CCT GTA C	CAC CTT GCA GCT CTT CCT GTA	HF544992
TaNPF7.2	CCC AGC AGT CAA AGC AAA CAC TG	GTA GCT GAA GAA GGC CAC CTT GG	HF544993

Figure S1. Average rainfall and max-min air temperature at Rothamsted field trails (ROTHMET : Rothamsted meteorological records www.era.rothamsted.ac.uk). **A.** Between 01.09.2006-31.08.2007. The time period from anthesis to complete leaf 2 senescence is grey shaded. **B.** Enlargement wheat var Hereward time period from anthesis to complete senescence showing detailed daily rain fall and max-min air temperature.

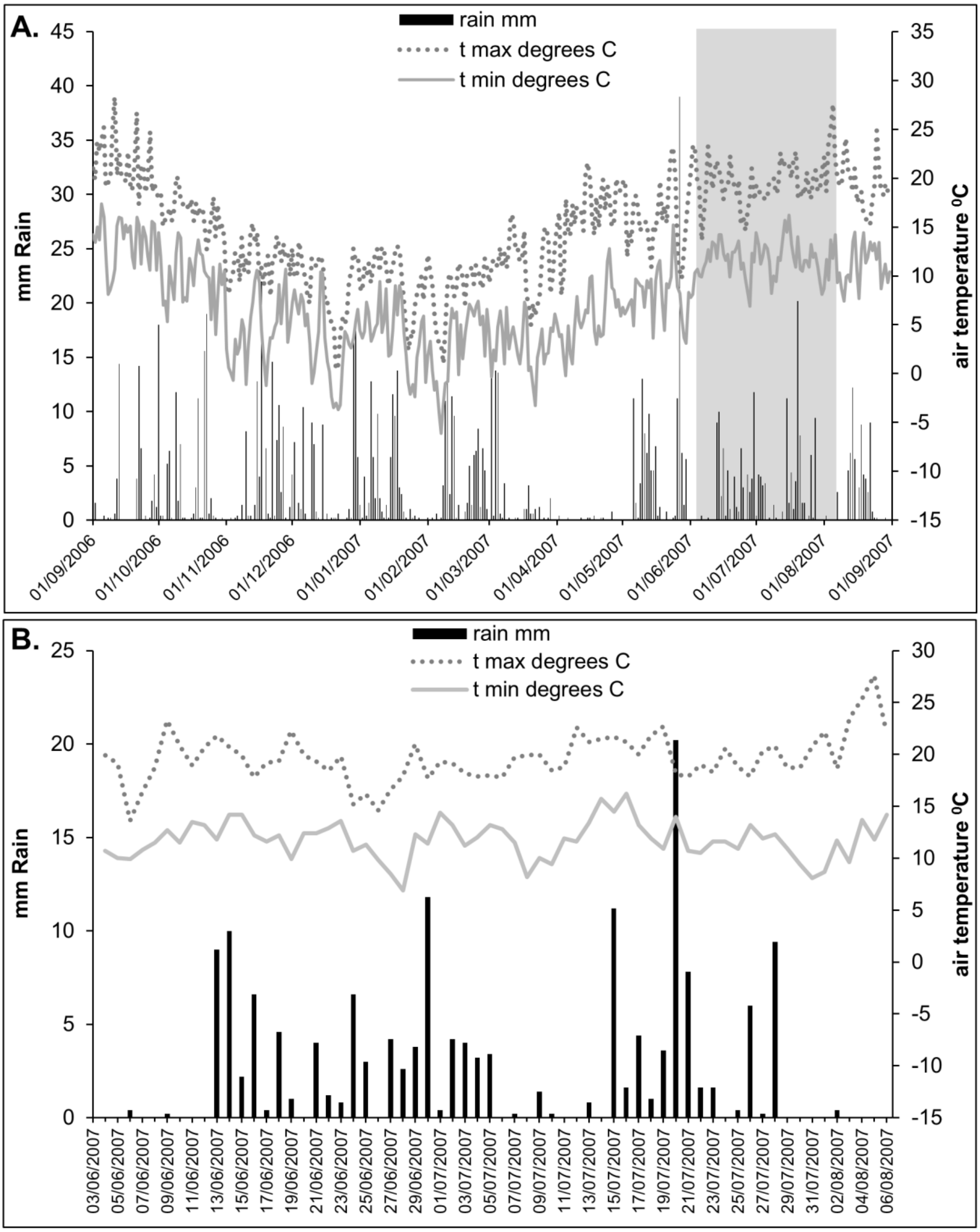

Supplement: Supplementary Data [file supp_eru231_Supplemental_data___Buchner.pdf]
